# Prediction of microbial communities for urban metagenomics using neural network approach

**DOI:** 10.1186/s40246-019-0224-4

**Published:** 2019-10-22

**Authors:** Guangyu Zhou, Jyun-Yu Jiang, Chelsea J.-T. Ju, Wei Wang

**Affiliations:** Department of Computer Science, University of California, Los Angeles, CA United States

**Keywords:** Urban metagenomics, Multi-label classification, Neural network

## Abstract

**Background:**

Microbes are greatly associated with human health and disease, especially in densely populated cities. It is essential to understand the microbial ecosystem in an urban environment for cities to monitor the transmission of infectious diseases and detect potentially urgent threats. To achieve this goal, the DNA sample collection and analysis have been conducted at subway stations in major cities. However, city-scale sampling with the fine-grained geo-spatial resolution is expensive and laborious. In this paper, we introduce MetaMLAnn, a neural network based approach to infer microbial communities at unsampled locations given information reflecting different factors, including subway line networks, sampling material types, and microbial composition patterns.

**Results:**

We evaluate the effectiveness of MetaMLAnn based on the public metagenomics dataset collected from multiple locations in the New York and Boston subway systems. The experimental results suggest that MetaMLAnn consistently performs better than other five conventional classifiers under different taxonomic ranks. At genus level, MetaMLAnn can achieve F1 scores of 0.63 and 0.72 on the New York and the Boston datasets, respectively.

**Conclusions:**

By exploiting heterogeneous features, MetaMLAnn captures the hidden interactions between microbial compositions and the urban environment, which enables precise predictions of microbial communities at unmeasured locations.

## Background

Metagenomics studies the genomic content obtained from a human body site or an environment with a goal of understanding microbial diversity. The microorganisms in our environment are greatly associated with human health and disease.

Human microbiome studies are already rich enough to uncover the microbial diversity within the human body [1]. Environmental metagenomics, though falling behind in the past years, has also become increasingly important due to the increasing awareness of its impacts on public health, especially in densely populated urban areas [2–8]. Therefore, the effectiveness of a city’s long-term disease surveillance and health management relies heavily on how we understand and predict the metagenomics composition at a fine-grained level.

Many recent research have been devoted to building up city-scale metagenomic profiles [9, 10]. For example, Afshinnekoo et al. [9] created a city-wide metagenomic profile for New York City by collecting samples from different surfaces across the entire New York subway system. Taxonomic assignments were generated by alignment reading, and the relative abundances were computed at the species level. The profile described the pattern of metagenomic communities and revealed how the human interacts with new microbes or danger pathogens. Another study conducted by Hsu et al. [10] provided a more comprehensive metagenomic profile in the Boston transportation system, which described microbial communities across multiple surface types. However, collecting, sequencing, and analyzing the metagenomics data at every station cost them a great amount of money and time. Given that, our study focuses on developing a model to automatically predict the microbial communities for unsampled locations.

**Fig. 1:**
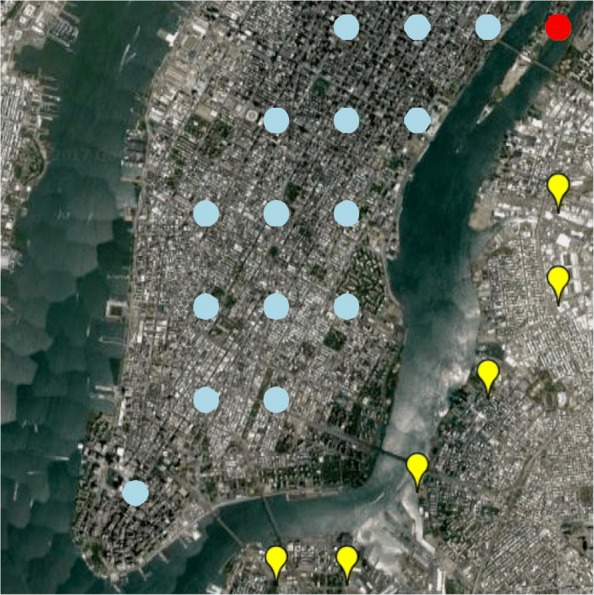
There are three groups of subway stations based on the hierarchical clustering of the microbial community abundance in each location. We set the number of clusters to be three and use the Pearson correlation as the distance metric. We observe that the East river is a clear boundary that separates the three districts: Manhattan (blue dots), Brooklyn (yellow marks), and the Roosevelt Island (one red dot at top right)

It is challenging to predict the microbial communities for unsampled locations. First, the characteristics of microbial communities can vary enormously in a complicated urban system due to various factors like geographical topology and public transit network. Many recent works have investigated how network connectivity affects the similarity of microbiomes. For examples, Leung et al. [2] conducted a Mantel test of Hong Kong subway line (MTR), and found that closely connected MTR lines shared more similar microbial communities than pairs that are further apart (*R*=0.47, *P*=0.03), probably because of distance-dependent dispersal and transferring commuters. To further evaluate the assumption, we conduct a clustering analysis based on microbial community similarity at different locations. As shown in Fig. 1, different microbes are separated by geographical boundaries.

Second, the formation and transmission of microbial communities are also affected by the material type of surfaces where the samples are collected [10]. Lastly, within each community, the genetic properties of each individual microorganisms and the correlation between individual microorganisms also contribute to the complexity. Considering the mixed effects from various factors, a simple model for each station along the same subway line should not be enough.

To address these challenges, we formulate the prediction of microbial communities at unsampled locations as a multi-label classification (MLC) task. Based on a set of heterogeneous features extracted from the urban environment, we aim to predict the presence or absence of a list of microbes at a nearby location. For MLC, each location is considered as an instance and each label represents a microbe.

Since different class labels have to be predicted simultaneously [11], MLC is suitable for solving the microbes inference problem, with their dependencies exploited at the same time. These properties reflect the nature of microbial communities.

In the field of urban computing, statistical models like regression trees have been applied to do real-time air quality prediction. For example, in U-Air [12], the authors inferred the fine-grained air quality in a city by using a semi-supervised learning approach. The model was able to predict air quality at non-monitored stations based on the air quality data reported by existing monitor stations. The spatial classifier for their model was based on an artificial neural network (ANN). However, this model only estimated a single value (i.e. the air quality index) for each location, so it was also inadequate to address the MLC task we formulated.

In the field of metagenomics, several computational models, such as BioMiCo [13] and NMF [14] have been developed to infer microbial community structures. To estimate the composition of each sample given the abundance profile, BioMiCo uses the supervised Bayesian model while NMF leverages the matrix factorization.

Nevertheless, these works cannot directly infer the microbial community for unsampled locations in the urban environment due to their inability to incorporate spatial information.

All the models mentioned above either cannot address the complicated environmental conditions or handle the intricate relationships between microbial compositions and the urban environment. In our recent work [15], we propose MetaMLAnn (**M**etagenomic **M**ulti **L**abel **A**rtifical **n**eural **n**etwork), a neural network based and supervised learning model to predict the microbial community for city-scale metagenomics. MetaMLAnn is built on the widely-used feed-forward neural network model. But unlike the conventional feed-forward neural network model that predicts each label individually, it leverages an extra shared structure to capture the dependencies among different labels (microbes). To begin with, we train MetaMLAnn using a state-of-the-art network embedding technique to integrate features constructed from different data sources. Next, we leverage manifold regularization to extend our model. Our model is robust to the sparse samples with limited labeled data by incorporating the domain knowledge. To further improve our model, we also introduce an ensemble model, MetaMLAnn+, which can outperform each individual model by leveraging the diversified information from MetaMLAnn and different classification models with the strong signal. To our best knowledge, our work is the initial attempt to predict the microbial community for urban metagenomics by using the neural network model. In this paper, we extend our previous work by presenting detailed theoretical foundations and additional statistical analyses.

We summarize the contribution of this paper as follows:

- This is the first series of in-depth study of microbial communities inference for unsampled locations. The inference task is formulated as a multi-label classification problem and a neural network learning technique (MetaMLAnn) is proposed to solve it.
- We integrate the manifold regularization into our framework to guide the training of MetaMLAnn. We provide detailed theoretical foundations of showing how the domain knowledge of microbial evolutionary relationships helps.
- Important features are extracted from multiple data sources, including city-scale transit features and surface material. An in-depth feature importance study has also been provided.
- We evaluate MetaMLAnn on the New York and Boston subway metagenomic DNA sequencing data samples. We present detailed discussions about that MetaMLAnn performs better against five baseline methods under two datasets with different level of the taxonomy. We also analyze the importance of using the ensemble model.

## Materials and methods

In this section, we present the detailed designed of our framework and describe the dataset used in this work.

### Preliminaries and problem definition

We start with formalizing the mathematical notations of our model. Table 1 summarizes the symbols we use in this article.

**Table 1:** Summary of symbols

Symbol	Description
*M*	An alphabetical ordered list of microbial names of identified organisms
*Y*_*i*_	A vector of microbial distribution given location *i*, where *Y*_*ij*_ indicates the existence of microbe *M*_*j*_
*Y*_*n*∗ *m*_	An *n* by *m* microbial distribution matrix, where *Y*_*ij*_ represents whether *M*_*j*_ exists in the location *i*
*F*	A *k*-dimensional feature vector
*X*_*n*∗ *k*_	An *n* by *k* feature matrix
*S*	A set of locations, where *s*_*i*_ is the *i*^*th*^ location
*P*_*m*∗ *m*_	An *m* by *m* pairwise evolutionary (phylogenetic) microbial similarity matrix

#### **Definition 1**

(Microbe Index) Microbe Index is defined as an alphabetically ordered list of microbial names of identified organisms. Each element in the list is a taxonomic name.

#### **Definition 2**

(Microbial Distribution Matrix) All samples at different locations are represented as a matrix *Y*∈*R*^*n*×*m*^, where *n* is the number of sampling locations, and *m* is the total number of microbes in the Microbe Index. Each row *Y*_*i*_ represents the microbial distribution vector of location *i*. Each element *Y*_*ij*_ represents whether the *j*^*th*^ microbe exists (or its relative abundance meets a threshold *γ*) in the *i*^*th*^ location. More specifically,

$$ Y_{ij} = \left\{\begin{array}{ll} 0 & Y_{ij}< \gamma\\ 1 & Y_{ij} \geq \gamma\\ \end{array}\right. $$

#### **Definition 3**

(Multi-Label Classification) Given $\mathcal {X} \in R^{n\times k}$, a set of *n* instances, each being a *k*-dimensional feature vector, and $\mathcal {Y} = \{y_{1}, y_{2}, \ldots, y_{m}\}= \{0,1\}^{m}$, a set of labels, where each element is 1 if the label is relevant and 0 otherwise.

The classification model is to learn an estimation function *f*:*R*^*k*^→2^*m*^ that assigns a subset of labels to a given instance.

In our microbial community inference case, we extract feature vectors of *n* samples and represent them as *X*. The Microbe Index created from known locations is used as *Y*, where the order of microbes is preserved.

**Problem statement.** Suppose *S*=*S*_1_∪*S*_2_={*s*_1_,*s*_2_,…,*s*_*n*_}, where *S*_1_ and *S*_2_ are sets of sampled and unsampled locations, respectively. Each sampled location *s*_*i*_∈*S*_1_ is associated with a microbial distribution vector $Y_{s_{i}}$. Our goal is to predict $Y_{s_{j}}$ of each *s*_*j*_∈*S*_2_, which is not sampled.

**Fig. 2:**
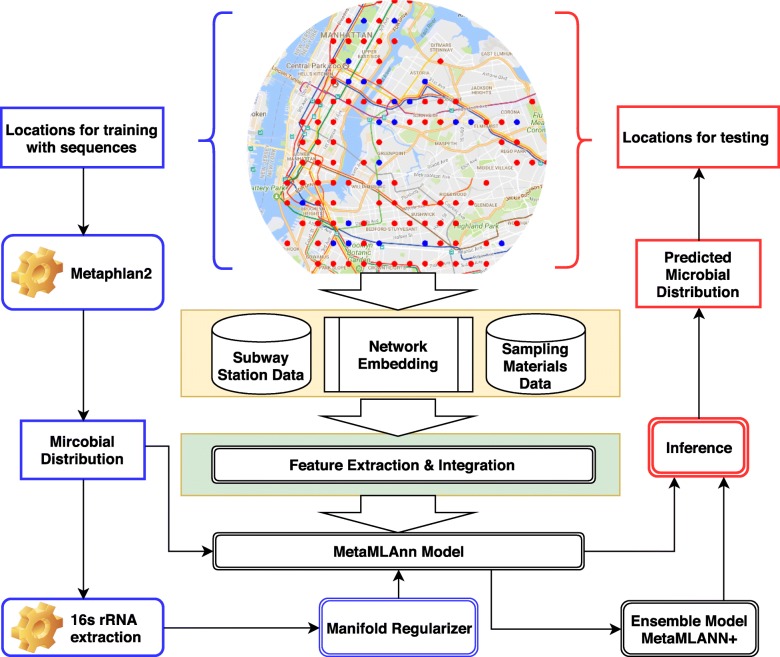
Our general framework. Starting from the map, we simulate the inference task by splitting the samples into the training set (blue dots) and test set (red dots). We use Metaphlan2 [16] to obtain the microbial distribution profiles from the raw sequencing data. We first extract and integrate features for both training and test data. We also construct the evolutionary (phylogenetic) microbial similarity matrix, using the 16s rRNA of the bacteria as a regularizer. Then, we feed the training data’s features and the similarity matrix into MetaMLAnn, which will perform microbial inference based on the features of test dataset. Our model can also be integrated with other classification models trained with same features as an ensemble model

The framework of MetaMLAnn is shown in Fig. 2. It contains two major components and one model: the blue component for learning and the red component for inference, together with the MetaMLAnn model. In the following subsections, we introduce how MetaMLAnn is constructed, explain the regularization framework, discuss how feature extraction has been done to train MetaMLAnn, and present the ensemble model.

### Model: MetaMLAnn

We start with introducing the one hidden layer feed-forward neural network model [17]. In the neural network model, there are *p* hidden units. The input layer *x*∈*R*^*k*×1^ is connected to hidden layer *h*∈*R*^*p*×1^ with weights *W*^(1)^∈*R*^*p*×*k*^ and biases *b*^(1)^∈*R*^*p*×1^. The hidden nodes are then connected to output nodes *o*∈*R*^*m*×1^ via weights *W*^(2)^∈*R*^*m*×*p*^ and biases *b*^(2)^∈*R*^*m*×1^.

We denote *f*_*θ*_:*x*→*o* as the feed-forward neural network below:

1 $$ f_{\theta}(x) = f_{o}\left(W^{(2)}f_{h}\left(W^{(1)}x + b^{(1)}\right) + b^{(2)}\right),  $$

where, *θ*={*W*^(1)^,*W*^(2)^,*b*^(1)^,*b*^(2)^}. *f*_*o*_ and *f*_*h*_ are activation functions in the output layer and the hidden layer respectively. Specifically, the function *f*_*θ*_(*x*) can be simplified by using vector representation as follows, where *z*^(1)^ and *z*^(2)^ are the vector representations of the weighted sums of inputs and hidden activation functions as follows:

2 $$\begin{array}{*{20}l} z^{(1)} &= W^{(1)}x + b^{(1)}, h = f_{h}\left(z^{(1)}\right), \end{array} $$

3 $$\begin{array}{*{20}l} z^{(2)} &= W^{(2)}h + b^{(2)}, o = f_{o}\left(z^{(2)}\right) \end{array} $$

Given the cost function *J*(*θ*;*x*,*y*), we seek for a parameter vector *θ* which minimizes it. *J*(*θ*;*x*,*y*) measures the difference of given targets *y* and predictions of the network. Here, we choose Cross-Entropy [18] as our cost function:

4 $$ J_{CE}(\theta;x,y) = -\sum_{i}(y_{i} \log o_{i}) + (1-y_{i})\log(1-o_{i}),  $$

where *y*_*i*_ and *o*_*i*_ are the ground truth and the predicted scores for label *i*, respectively. The sigmoid activation function *o*=*σ*(*z*)=*f*_*o*_(*z*)=1/(1+*exp*(−*z*)) is applied in the output layer.

**Fig. 3:**
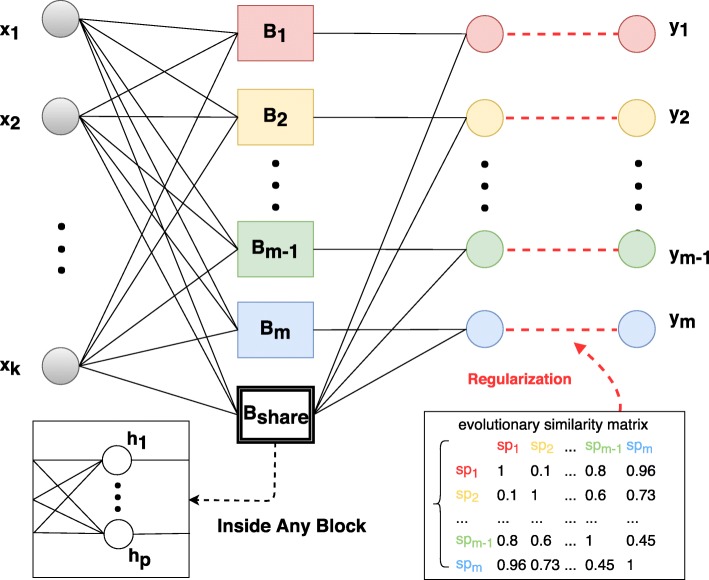
The architecture of our proposed model MetaMLAnn. Starting from the left, the input layer with *k* nodes will receive *k* features respectively. Then, every input node will connect to all blocks, where each block contains *p* hidden units. The shared block (*B*_*share*_) is connected to every output label, and every other individual blocks (*B*_1_…*B*_*m*_) is connected to its corresponding output label. It is regularized by the evolutionary (phylogenetic) microbial similarity matrix

In MetaMLAnn, we extend the basic form feed-forward neural network by leveraging a heterogeneous architecture. Figure 3 depicts the detailed design of MetaMLAnn. Instead of using multiple hidden nodes of the same type in the hidden layer, we denote two different types of sub hidden layers which we call blocks (*B*). The first set of blocks are called individual blocks, *B*_1_ to *B*_*m*_ where *m* is the number of labels. The second type of block, *B*_*share*_, is a shared block that connects to all output neurons. Therefore, each output neuron connects to a corresponding individual block and a commonly shared block. All blocks contain one hidden layer with *p* neurons.

Therefore, we replace the *p* units hidden layer with *m*+1 blocks *B*. Each block consists of a hidden layer with *p* hidden neurons. For each *i*, the input layer *x*∈*R*^*k*×1^ is connected to each block *B*_*i*_∈*R*^*p*×1^ with weights $W_{i}^{(1)} \in R^{p\times k}$ and biases $b_{i}^{(1)} \in R^{p\times 1}$. Then, the blocks *B*_*i*_ and *B*_*share*_ are connected to output node *o*_*i*_∈*R* via weights $W_{i}^{(2)} \in R^{1\times p}$ and biases *b*^(2)^∈*R*.

**Fig. 4:**
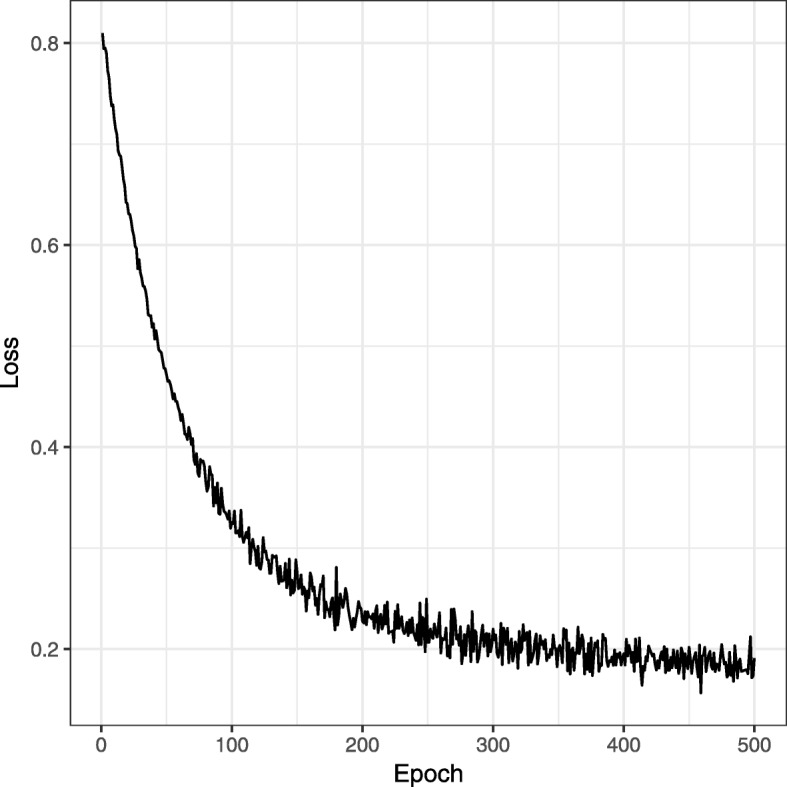
The values of the loss function over different numbers of optimization epochs with the New York dataset

We use stochastic gradient descent (SGD) [19] to efficiently optimize the cost function in Eq. 4. We randomly sample a location *i* and a unit from *y*_*i*_ to compute *B*_*i*_ for each individual block. We randomly sample a location *i* and a unit from all the classes among *y*_1_ and *y*_*m*_ to capture the global properties shared by all microbes for the shared block *B*_*share*_,. The updating rules for different variables *W* and *b* can be derived by taking the derivatives of the above objective function and applying SGD. Training our model is efficient with SGD and back-propagation. More specifically, the time complexity of training our model is *O*(*t*·*n*·∣*θ*∣), where *t* is the number of training epochs; *n* is the number of training examples; *θ* is the set of parameters in the model. To demonstrate the convergence of the proposed algorithm, we plot the values of the loss function over different optimization epochs in Fig. 4.

Finally, the heterogeneous neural network model *f*_*θ*_:*x*→*o* can be reformatted as follows:

$${}{\begin{aligned} z^{*(1)} &= \left[z_{1}^{(1)}, \ldots, z_{m+1}^{(1)}\right],\text{where}\ z_{i}^{(1)} = W_{i}^{(1)}x + b^{(1)}, \\ B^{*} &= f_{B}\left(z^{*(1)}\right) = [B_{1}, \ldots, B_{m+1}], \text{where}\ B_{i} = f_{B}\left(z_{i}^{(1)}\right), \\ z^{*(2)} &= \left[z_{1}^{(2)}, \ldots, z_{m+1}^{(2)}\right], \text{where} \\ z_{i}^{(2)} &= W_{i}^{(2)}B_{i} + W_{m+1}^{(2)}B_{m+1} + b^{(2)}, o = f_{o}\left(z^{*(2)}\right)\\ \end{aligned}} $$

### Manifold regularization

Neural networks tend to suffer from limited training examples. However, with only a few instances of each label, it is challenging to train MetaMLAnn. One potential solution to compensate for the data sparsity is to incorporate prior knowledge. Inspired by the general observation that evolutionary relationships are expected to be associated with patterns of community composition [20], we presume that the groups of microbes tend to co-occur in the same community when they are closely related to each other in the taxonomy.

The taxonomy here is referred as the identification, naming, and classification of organisms. We choose to use the evolutionary similarity as the domain knowledge, which is then fed into our regularizer. This is because taxonomy is often informed by the evolutionary relationships among different microbes (i.e., phylogenetic). To incorporate such microbial similarity, many regularization techniques can be used. We choose one of the most popular techniques, Graph Laplacian regularizer, to build our regularization frameworks [21–25].

#### **Definition 4**

(Graph Laplacian matrix *L*) Given a pairwise similarity matrix *P*∈*R*^*m*×*m*^, the Graph Laplacian matrix is defined as *L*=*D*−*P*, where *D* is a diagonal matrix with *j*^*th*^ diagonal element $D_{j,j} = \sum _{j' =1}^{m}\left (P_{j,j'}\right).$

By minimizing

5 $$  \Omega(\beta) = \frac{1}{2} \sum_{1 \leq i, i' \leq I} P_{i, i'} \left\| \beta_{i} - \beta_{i'}\right\|^{2}_{2},  $$

the regularizer can preserve the local geometrical structure of a parameter vector *β* with length *I*. According to the definition, we observe that *L* has the following property that makes it suitable for regularization. Given the trace operator *tr*(·):

6 $$\begin{array}{*{20}l} \Omega(\beta) &= \sum_{1 \leq i, i' \leq I} P_{i, i'} \beta_{i}^{T} \beta_{i} - \sum_{1 \leq i, i' \leq I} P_{i, i'} \beta_{i}^{T} \beta_{i'}  \\ &= tr\left(\beta^{T} D \beta\right) - tr\left(\beta^{T} P \beta\right) = tr\left(\beta^{T} L \beta\right) \end{array} $$

From the above equations, the two parameters *β*_*i*_ and $\beta _{i'}\phantom {\dot {i}\!}$ are enforced to be similar, which can be incorporated into the cost function. The regularized cost function is defined as:

7 $$ {}{\begin{aligned} J_{CE_{reg}}(\theta;x,y) &= -\sum_{i} \left[ \left(y_{i} \log o_{i}\right) + \left(1-y_{i}\right)\log\left(1-o_{i}\right)\right] \\&\quad+ \lambda tr\left(\beta^{T} L \beta\right), \end{aligned}}  $$

where *y*_*i*_ and *o*_*i*_ are the ground truth label and the predicted score for sample *i*.

The Graph Laplacian regularizer can represent any pairwise relationships between parameters. Here we discuss how to use the evolutionary similarities as priors and the corresponding Laplacian regularizers to incorporate structured domain knowledge. The Laplacian matrix *L* is firstly obtained by constructing the pairwise evolutionary similarity matrix (*P*) of different microbes.

Upon obtaining the predicted microbial distribution vector $Y_{i}^{*}$ for given location *i* from the blocks, each vector is regularized by feeding $Y_{i}^{*}$ into Eq. 5, where *β* refers to the predicted vector $Y_{i}^{*}$ and *β*_*i*_, *β*_*j*_ refers to microbe *i* and microbe *j* at this location, respectively.

### Feature extraction

Here we describe how we extract the features from various data sources. These feature extraction methods can serve as a general pipeline for any urban-scale metagenomics study.

We define a feature vector as *F*:*R*^*k*^, where *R* is a *k* dimensional feature.

For this work, we extract the following features: subway station information, inter-station connections, and sampling surface materials. All features are concatenated into a feature vector for each sample and are used to train MetaMLAnn.

**Subway station features (*F***_***s***_**):** The first set of features that we extracted is the subway station information. We obtain the MTA and MBTA subway station data for New York and Boston. Each location is associated with the closest stations within a predefined radius, *r*=0.01 miles. This radius value is an empirical parameter and can be tuned. The feature vector is then created based on the lines that pass through the current station. If there is no station information available in this range, we will find the 2 nearest stations and see if their subway line information matches. If they do match, we will align the subway line to this location. Otherwise, we will not assign any subway line information to this location. This process is specifically for dealing with sampling locations which are not stations, but in between two subway stations on the same line.

It has been shown that the number of riders is positively correlated with the amount of DNA collected in a station [9]. Therefore, we also retrieve the public MTA data with the turnstiles usage information at each station. The corresponding node vector is then weighted by the average number of riders within DNA collection date at each station.

For example, there are in total 25 different subway lines in New York, thus we create a binary vector of size 25, each element in the vector indicates whether this line will pass this location or not. For example, for station *l*, the subway line feature vector is defined as $F_{s_{l}} = (v_{1}, v_{2}, \ldots v_{25})$. If *v*_*i*_=1, then line *i* passes through this location. Finally, $F_{s_{l}} $ will be weighted based on the busyness of station *l*.

Note that it is possible one location is associated with multiple lines or no lines. For the multiple lines’ case, there will be more than one *v*_*i*_ equal to 1. For the case of no line, we will simply remove such location since we focus on the inference at stations. Therefore, all locations will be associated with a subway line feature as a vector.

**Interconnection features (*F***_***c***_**):** We first describe how we construct the subway system network. Each subway station is denoted as a node and each interaction between two stations is drawn an edge. The weight of edge (*i*,*j*) is computed by the minimum number of stops from station *i* to station *j*. We also consider the case of express trains and if there exist express trains directly connecting two stations, we assign 1 as the weight to that edge.

Upon obtaining the station network, we apply the network embedding algorithm Node2Vec [26]. Each node is embedded into a low dimensional vector based on the generated network.

**Surface materials features (*F***_***m***_**):** The surface materials are strongly correlated with the microbial communities, as discussed in [10]. Therefore, we represent such information by using another set of vectors. Based on the type of materials it was collected from, a vector of length equal to the number of material types is constructed. For the New York dataset, each element represents one type of material: ‘concrete’, ‘metal’, ‘plastic’,‘water’ or ‘wood’ and the vectors are of length 5. As for the Boston dataset, the vector is of length 4 with four types of materials: ‘glass’, ‘polyester’, ‘PVC’, and ‘steel’.

### Ensemble with hybrid prediction

To alleviate the lack of training data, in addition to the regularization, we also propose to construct an ensemble of MetaMLAnn with any other model that needs fewer training samples.

For each label *i*, let *o*_*i*_ be the predicted score of MetaMLAnn. Given the score from the other model *m* as $o^{m}_{i}$, we conduct a linear hybrid prediction for ensemble as follows:

8 $$ o^{h} = \alpha\cdot o_{i} + \left(1 - \alpha\right) o^{m}_{i},   $$

where 0≤*α*≤1 is a parameter to decide the weights of two models. When *α*=1 the prediction is MetaMLAnn, and when *α*=0 the prediction is the model *m*.

We denote the ensemble approach as MetaMLAnn+.

### Sample collection and data preprocessing

We apply our model on the New York and Boston datasets obtained from the MetaSUB Inter-City Challenges track of the 2017 CAMDA Contest.

They both contain mass-transit metagenomic raw reads data, supplemented with sample descriptions.

The New York dataset contains 1572 samples, representing different sites. These samples were collected from open subway stations for all 24 subway lines of the NYC Metropolitan Transit Authority (MTA). At subway stations, samples were collected in triplicate, with one sample taken inside a train at the station and two samples from the station itself, as reported by [9]. DNA samples collected from each site were sequenced using Illumina platform, with a total of 10.4 billion paired-end DNA sequencing reads.

In addition, each sample is also associated with meta information, including the latitude and longitude showing where the sample was collected, and surface materials. All these information are indispensable for the enrichment of feature generation.

Similarly, there are 141 samples in the Boston dataset, which have been also collected from the local subway system, consisting of 5 lines (red, orange, blue, green, and silver) that extend from downtown Boston into the surrounding suburbs. As mentioned in [10], most samples are 16S rRNA gene amplification sequence data, and a subset of the samples are subjected to shotgun metagenomic sequencing. Each sample is also supplemented with additional information, which describes the date of collection, station information, and surface type. For the 16S rRNA samples, the corresponding abundances profiles are also provided.

For each sample in the New York dataset and samples subjected to shotgun metagenomic sequencing in the Boston dataset, we conduct the following preprocessing steps:

1. To be consistent with the processing procedure in [9] from which the New York data is collected, We use MetaPhlan2 [16] to perform microbial profiling. Each profile contains the relative abundances as a percentage from the kingdom level to the species level.
2. There are 48.3% of the reads that do not match to any known organism in the New York dataset, as described in [9]. Therefore, when we construct the microbial distribution vector, those unknown microbes are removed and the relative abundances of the remaining known microbes are recomputed.

### Supplemental data sources

We use the New York subway station data and the Boston subway station data from the MTA and MBTA website respectively to construct the subway line features. They contain geographic locations, subway station names, and subway lines that pass each station. We also obtain the turnstile data of MTA and MBTA to count the busyness of all stations. The detailed descriptions can be found in Table 2.

**Table 2:** Supplemental data sources

Data	Description	Reference
MetaSub	Metagenomic subway station datasets for New York and Boston	http://camda2017.bioinf.jku.at/doku.php/contest_dataset
MTA	New York subway station and lines	http://web.mta.info/ developers/data/nyct/ subway/Stations.csv
MBTA	Boston subway station and lines	https: //d3044s2alrsxog. cloudfront.net/sites/ default/files/2017-11/ subway-1.txt
Turnstile of MTA	Turnstile entry and exit of MTA	http://web.mta.info/ developers/turnstile. html
Turnstile of MBTA	Turnstile entry and exit of MBTA	https://github.com/ mbtaviz/mbtaviz. github.io/releases/ download/data/ turnstile_data.csv.gz

To capture the underlying microbiota structure, we construct a pairwise similarity matrix to represent the evolutionary relationship between two species. We retrieve the 16S ribosomal RNA sequence for bacteria and archaea, 5S ribosomal RNA for eukaryotes, and the whole DNA sequences for viruses from the NCBI [27–29] and the Silva [30, 31] database. We perform sequence alignments to compute the pairwise similarity within each kingdom. We normalize the similarity values to the range of 0 to 1 and we assign 0 to their similarity for cross-kingdom species pairs. Finally, we take the mean of all species’ similarity scores under that level and aggregate them as the new score for each genus pairs (Eq. 9). In this way, we can obtain the similarity matrix between genus level.

Given two genus *g*_*a*_ and *g*_*b*_ as sets of species, the similarity score between the pair of genus can be computed as:

9 $$  sim(g_{a}, g_{b}) = \frac{1}{|g_{a}|\cdot |g_{b}|} \sum_{sp_{a} \in g_{a}} \sum_{sp_{b} \in g_{b}} sim(sp_{a}, sp_{b}),  $$

where *sp*_*a*_ and *sp*_*b*_ are the species of *g*_*a*_ and *g*_*b*_, respectively.

## Results

To demonstrate the effectiveness of MetaMLAnn, we conduct comprehensive experiments by using both the New York and Boston datasets. In this section, we will discuss the experiment setup, evaluation metrics, baselines and results.

### Experimental settings

After we conduct data processing, each sample is associated with an abundance vector.

It is observed that many species are seriously under-represented (i.e. appearing at only one location) for the abundance at all levels. We choose to focus on the genus-level abundance to alleviate the issues including under-represented microbes, missing species-level taxonomy, and very similar microbial species.

Together with the number of features obtained, the detailed microbial composition of both dataset can be found in Table 3.

**Table 3:** Description of New York and Boston datasets

		New York	Boston
Number of features		46	43
Number of labels (Microbes)	Bacteria	232	209
	Eukaryotes	15	7
	Archaea	8	5
	Viruses	14	15

Number of features obtained and number of labels at genus level, grouped by four different kingdoms

We use *k*-fold cross-validation for all experiments. Setting the value of *k* to be three, we randomly and equally split the data into three non-overlapping subsets. Each subset has a chance to train the model and to test the model.

The average performance of each method from these three folds is reported. In addition, we also justify the effectiveness of our feature construction by comparing the performance of individual features and their combination with the same classifier.

### Evaluation metrics

We assess the performance of our classifier in several ways. While accuracy is the simplest and the most straightforward measure, it is biased toward classes with a larger sample size. Instead, we report precision, recall, and F1 score as our evaluation metrics. These metrics are defined as:

10 $$\begin{array}{*{20}l} \text{precision} &= \sum_{i=1}^{m} tp_{i}/\left(\sum_{i=1}^{m} tp_{i} + \sum_{i=1}^{m} fp_{i}\right) \end{array} $$

11 $$\begin{array}{*{20}l} \text{recall} &= \sum_{i=1}^{m} tp_{i}/\left(\sum_{i=1}^{m} tp_{i} + \sum_{i=1}^{m} fn_{i}\right) \end{array} $$

12 $$\begin{array}{*{20}l} \text{F1 score} &= \frac{2*precision*recall}{precision+recall} \end{array} $$

where given *m* labels, *tp*_*i*_, *tn*_*i*_, *fp*_*i*_ and *fn*_*i*_ represents true positives, true negatives, false positives and false negatives for *i*^*t*^*h* label respectively.

Finally, we also use ranking loss, which averages over *n* samples the number of label pairs that are incorrectly ordered, i.e. true labels have a lower score ($\hat {f}$) than false labels, weighted by the inverse number of false and true labels, as shown below:

13 $$ \begin{aligned} ranking loss &= \frac{1}{n} \sum_{i = 1}^{n} \frac{1}{|y_{i}|(m-|y_{i}|)} \left|\{(k,l):\hat{f}_{ik} < \hat{f}_{il}, y_{ik}\right.\\ &\left.= 1, y_{il} = 0\}\right| \end{aligned}  $$

### Baselines

As we formalize the inference problem as a multi-label classification (MLC) problem, we adopt several widely used MLC algorithms as the baseline methods, including Inverse Distance Weighting (IDW) interpolation, *k* Nearest Neighbor (kNN) [32], Support Vector Machine (SVM) [33], Random Forest [34], and Neural Network [35].

- Inverse Distance Weighting (IDW): Inverse distance weighting is a deterministic, nonlinear interpolation technique that uses a weighted average of the attribute values from nearby sample points to estimate the magnitude of that attribute at non-sampled locations. The weight a particular point is assigned depends upon the sampled point’s distance to the non-sampled location.
- K-Nearest Neighbor: This classifier will compute classification from a simple majority vote of the nearest neighbors of each point: a query point is assigned the data class which has the most representatives within the nearest neighbors of the point.
- SVM with one-vs-all: This baseline assumes all the label prediction are independent. Binary decomposition is used, on each binary classification task (one for each label). SVM is used as the base classifier. Then the one-vs-all is used, which consists of fitting one classifier per class. For each classifier, the class is fitted against all the other classes. Then the predictions of SVMs for all labels are combined to make the final prediction.
- Random Forest: This baseline method is an ensemble of decision tree classifiers. Based on various sub-samples of the dataset random forest will use averaging to improve the predictive accuracy and control over-fitting. In this baseline, we feed all the features equally into a decision tree.
- Single-layer Perceptron classifier (Vanilla Neural Network): We choose the single-layer feed-forward neural network model in the experiments for its simplicity and generality. It is the most similar classification model as MetaMLAnn.

### Performance of MetaMLAnn

Using the combined features, Tables 4 and 5 show the performance of MetaMLAnn and other aforementioned baselines on New York and Boston datasets, respectively. As discussed in experimental settings, we focus on the genus level inference. We observe that MetaMLAnn and MetaMLAnn+, outperform all baselines on F1 score and ranking loss.

**Table 4:** Evaluation of all the methods by cross validation on New York dataset at genus level

Evaluation metric				
Methods	Precision	Recall	F1 score	Ranking loss
IDW	0.5669	**0.6686**	0.6129	0.1790
kNN	0.7203	0.5109	0.5977	0.1273
SVM	**0.7510**	0.4787	0.5845	0.0725
Random Forest (RF)	0.7288	0.5026	0.5941	0.1365
Neural Network	0.7419	0.5110	0.6050	0.0718
MetaMLAnn	0.7456	0.5325	**0.6212**	**0.0682**
MetaMLAnn+ IDW	0.6578	0.6170	**0.6363**	**0.0688**

Higher precision, recall, F1 score, and lower ranking loss indicate better performance. Bold entries indicate best performance among different methods

**Table 5:** Evaluation of all the methods by cross-validation on Boston dataset at genus level

Evaluation metric				
Methods	Precision	Recall	F1 score	Ranking loss
IDW	0.5316	0.6177	0.5691	0.1929
kNN	0.7359	0.6266	0.6723	0.1837
SVM	0.7583	0.5366	0.6282	0.1473
Random Forest (RF)	0.7318	0.6214	0.6682	0.1630
Neural Network	0.7228	0.5594	0.6214	0.1297
MetaMLAnn	**0.7674**	**0.6706**	**0.7095**	**0.1270**
MetaMLAnn+ RF	**0.7744**	**0.6862**	**0.7229**	**0.1283**

Higher precision, recall, F1 score, and lower ranking loss indicate better performance. Bold entries indicate best performance among different methods

In the New York dataset, MetaMLAnn and MetaMLAnn+ perform the best in terms of F1 score and ranking loss, though the precision and recall of MetaMLAnn rank second among other baselines. IDW achieves the highest recall but its precision is the lowest, which offsets its high recall. As an unsupervised learning model using the inverse distance weighting of surrounding microbial distribution vectors, IDW tends to predict more microbes than others. However, most of them are false positives. On the other hand, SVM shows a slightly higher precision than all methods but results in a poor recall. This implies that SVM based methods tend to be conservative in predicting the “presence” of species, which do not meet our expectation. MetaMLAnn tends to have the best balance of both precision and recall, which results in the best overall F1 score. In addition to MetaMLAnn, we also report the result of the ensemble model with IDW where we use *α*=0.7 as MetaMLAnn+ after parameter tuning.

As can be seen from the table, the F1 score can be further boosted by more than 1%, which is better than either of the single model.

As for the Boston dataset, our model outperforms all the baseline models in terms of precision, F1 score and ranking loss. Even though Random Forest achieves a bit higher recall than our model, its precision suffers from the issue of predicting too many microbes. However, after we leverage the Random Forest model as part of our ensemble model with the same parameter as New York, *α*=0.7, MetaMLAnn+ achieves the best score in all metrics against other baselines.

## Discussion

### Feature analysis

As feature extraction is crucial for inferring microbial communities in a complicated urban system with heterogeneous data sources, we first demonstrate the effectiveness of our feature construction. Recall that we have three groups of features: subway station features (*F*_*s*_), interconnection features (*F*_*c*_), and surface material features (*F*_*m*_). As shown in Table 6, a random forest model is used to compare the performance of individual features and their combinations. Overall, the complete features set have the best performance in precision, F1 score, and ranking loss. Note that we intentionally choose to use Random Forest instead of our model, MetaMLAnn, to conduct experiments. This is to demonstrate that our feature extraction techniques are beneficial in general to the microbial community inference problem without favoring our model.

**Table 6:** Performance of random forest using different feature set at genus level

Evaluation metric				
Features	Precision	Recall	F1 score	Ranking loss
*F*_*s*_+*F*_*m*_+*F*_*c*_	**0.7288**	0.5026	**0.5941**	**0.1365**
*F*_*s*_+*F*_*c*_	0.7285	0.4654	0.5679	0.1422
*F*_*s*_+*F*_*m*_	0.6751	**0.5283**	0.5927	0.1649
*F*_*c*_+*F*_*m*_	0.6930	0.5113	0.5861	0.1440
*F*_*c*_	0.7063	0.4611	0.5576	0.1376
*F*_*s*_	0.6498	0.5227	0.5791	0.1855
*F*_*m*_	0.6328	0.5258	0.5725	0.2552

Higher precision, recall, F1 score, and lower ranking loss indicate better performance. Bold entries indicate best performance among different methods

### Analysis on different taxonomic levels

To further demonstrate the generality of our model, we compare the performance of MetaMLAnn with other aforementioned baselines under different taxonomic levels from phylum to species. We ignore Kingdom level due to few numbers of classes.

**Fig. 5:**
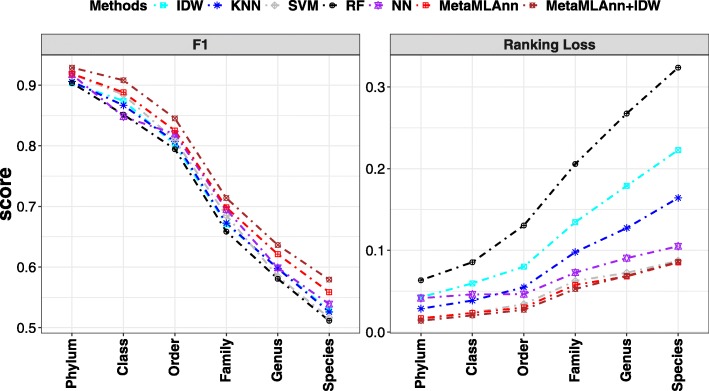
The performance of all approaches on the New York dataset over different metrics at different taxonomic ranks

As seen in Fig. 5, with the level of taxonomy becoming more specific, the performances of all methods decrease due to the increase of complexity. Against all competitors, MetaMLAnn and MetaMLAnn+ IDW constantly achieve the highest F1 score and the lowest ranking loss across all taxonomic levels. The advantage of MetaMLAnn becomes more obvious with a finer granularity of taxonomic level.

### Parameter selection of the ensemble model

Here, we analyze how the ensemble weight *α* affects the prediction performance.

**Fig. 6:**
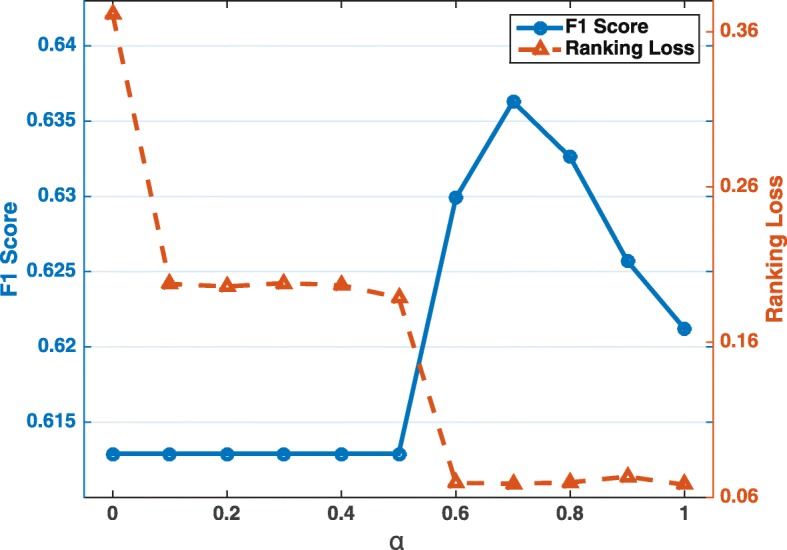
The F1 score and ranking loss performance on the New York dataset at genus level for the ensemble model MetaMLAnn+ that aggregates MetaMLAnn and IDW over different weights *α*

Figure 6 shows the F1 score and the ranking loss over different ensemble weights *α* of MetaMLAnn and IDW under the New York dataset. On the left vertical axis, we have F1 score (the larger the better) and on the right vertical axis, we have the ranking loss (the smaller the better). Recall that our ensemble model is defined in Eq. 8, where alpha closer to 1 means more weight on MetaMLAnn and closer to 0 means more weight on the additional model. The results suggest that with a good mixture of two models (i.e. *α*=0.7 for this case), the ensemble model can achieve the best for both F1 score and ranking loss. This is because the additional model (IDW) contains orthogonal information, which can compensate for the missing information from the training of MetaMLAnn. Without the ensemble model, MetaMLAnn tends to be conservative due to the sparsity of dataset. On the contrary, IDW tends to predict more microbes, which boosts the overall performance.

### Ablation study of the shared block *B*_*shared*_

Table 7 shows the results of the ablation study of the shared block *B*_*shared*_ and individual blocks *B*_*i*_, where *i*=1…*m*. *B*_*shared*_+*B*_*i*_. In the New York dataset, removing the shared block slightly decrease the F1 score and increase the loss while using only the shared block will downgrade the F1 score by around 3% and double the ranking loss. In the Boston dataset, dropping any of the two units largely impair the performance of MetaMLAnn. These results reflect the importance of having both the individual and shared hidden blocks in our model for predicting microbial communities.

**Table 7:** The results of ablation study of using different components of MetaMLAnn by cross validation on New York and Boston datasets at genus level

Evaluation metric				
	Precision	Recall	F1 score	Ranking loss
New York dataset				
*B*_*shared*_ only	0.7388	0.4986	0.5952	0.1299
*B*_*i*_ only	0.7339	**0.5379**	0.6204	0.0765
*B*_*i*_+*B*_*shared*_	**0.7456**	0.5325	**0.6212**	**0.0682**
Boston dataset				
*B*_*shared*_ only	0.6002	0.5920	0.5890	0.1896
*B*_*i*_ only	0.7574	0.5660	0.6428	0.1316
*B*_*i*_+*B*_*shared*_	**0.7674**	**0.6706**	**0.7095**	**0.1270**

Higher precision, recall, F1 score, and lower ranking loss indicate better performance. Bold entries indicate best performance among different methods

## Conclusions

Profiling city-scale microbial diversity is important for urban long-term disease surveillance and health management. The great efforts to collect DNA samples in densely populated cities still cannot meet the challenge to obtain the metagenomic profiles at fine-grained geo-spatial resolutions. To address this issue, we first define the task of inferring microbial community for city-scale metagenomics as a multi-label classification problem. We then propose MetaMLAnn, a neural network based approach to infer microbial communities of unsampled locations given the information from multiple data sources in the urban environment, including subway line information, sampling materials, and microbial compositions in sparsely sampled locations. The model captures the interactions between microbes and the urban environment by a shared hidden layer, and fuses the heterogeneous urban transit information with embedding for feature extraction.

Additionally, by incorporating signals from other strong models, the ensemble technique MetaMLAnn+ further improves the performance of the model. Extensive experiments demonstrate the effectiveness of our approach. In this work, we mainly focus on New York and Boston subway stations due to the limitation of data availability. In the future, with more cities being sampled, we plan to extend our model to the regional scale to solve the inter-city metagenomic inference problem.
